# Predicting informal dementia caregivers’ desire to institutionalize through mining data from an eHealth platform

**DOI:** 10.1186/s12877-024-05128-5

**Published:** 2024-08-30

**Authors:** Soraia Teles, João Viana, Alberto Freitas, Óscar Ribeiro, Sara Alves, Ana Ferreira, Constança Paúl

**Affiliations:** 1 Department of Behavioral Sciences, School of Medicine and Biomedical Sciences, University of Porto (ICBAS-UP), Rua de Jorge Viterbo Ferreira, 228, Porto, 4050-313 Portugal; 2 Center for Health Technology and Services Research at Health Research Network (CINTESIS@RISE), Rua Dr. Plácido da Costa, Porto, 4200-450 Portugal; 3 Department of Community Medicine, Information and Health Decision Sciences (MEDCIDS), Faculty of Medicine, University of Porto, Rua Dr. Plácido da Costa, Porto, 4200-450 Portugal; 4 Department of Education and Psychology, University of Aveiro, Aveiro, 3810-198 Portugal; 5 Santa Casa de Misericórdia de Riba D’Ave/CIDIFAD - Centro de Investigação, Diagnóstico, Formação e Acompanhamento das Demências, Tv. Conde de Riba de Ave, Riba d’Ave, 4765-288 Portugal

**Keywords:** Dementia, Informal caregivers, Desire to institutionalize, Platform data, eHealth, Classification tree

## Abstract

**Background:**

Dementia is a leading factor in the institutionalization of older adults. Informal caregivers’ desire to institutionalize (DI) their care recipient with dementia (PwD) is a primary predictor of institutionalization. This study aims to develop a prediction model for caregivers’ DI by mining data from an eHealth platform in a high-prevalence dementia country.

**Methods:**

Cross-sectional data were collected from caregivers registering on isupport-portugal.pt. One hundred and four caregivers completed the Desire to Institutionalize Scale (DIS) and were grouped into DI (DIS score ≥ 1) and no DI (DIS score = 0). Participants completed a comprehensive set of sociodemographic, clinical, and psychosocial measures, pertaining to the caregiver and the PwD, which were accounted as model predictors. The selected model was a classification tree, enabling the visualization of rules for predictions.

**Results:**

Caregivers, mostly female (82.5%), offspring of the PwD (70.2), employed (65.4%), and highly educated (M 15 years of schooling), provided intensive care (Mdn 24 h. week) over a median course of 2.8 years. Two-thirds (66.3%) endorsed at least one item on the DIS (DI group). The model, with caregivers’ perceived stress as the root of the classification tree (split at 28.5 points on the Zarit Burden Interview) and including the ages of caregivers and PwD (split at 46 and 88 years, respectively), as well as cohabitation, employed five rules to predict DI. Caregivers scoring 28.5 and above on burden and caring for PwD under 88 are more prone to DI than those caring for older PwD (rules 1–2), suggesting the influence of expectations on caregiving duration. The model demonstrated high accuracy (0.83, 95%CI 0.75, 0.89), sensitivity (0.88, 95%CI 0.81, 0.95), and good specificity (0.71, 95%CI 0.56, 0.86).

**Conclusions:**

This study distilled a comprehensive range of modifiable and non-modifiable variables into a simplified, interpretable, and accurate model, particularly useful at identifying caregivers with actual DI. Considering the nature of variables within the prediction rules, this model holds promise for application to other existing datasets and as a proxy for actual institutionalization. Predicting the institutional placement of PwD is crucial for intervening on modifiable factors as caregiver burden, and for care planning and financing.

## Introduction

Dementia represents a notable public health challenge, given its high prevalence and considerable economic and social ramifications for families, healthcare systems, and society at large [1]. Globally, an estimated 55 million individuals are affected by dementia, with 9.9 million new cases reported each year, including 2.5 million in Europe alone [2]. Portugal stands out among Organisation for Economic Co-operation and Development (OECD) countries, ranking fourth in dementia prevalence, following Japan, Italy, and Greece, with 21 cases of dementia per 1000 inhabitants [3].

Most individuals with dementia (PwD) prefer to age at home, where they can benefit from familiar environments and social connections, which has been linked to enhanced health-related quality of life [4, 5]. However, among older adults worldwide, dementia is a major cause of disability and dependency [6]. Studies from both high-income and low-and middle-income countries (e.g., [7–9]), converge on the conclusion that dementia makes the largest contribution to care needs among older people, surpassing other chronic diseases. Furthermore, dementia is a primary reason for institutionalization, with around 20% of PwD being placed in institutional care within one year of diagnosis, and admission rates escalating to nearly 90% within eight years [4].

Considering the health and social support infrastructure, institutional care poses notable financial burdens owing to its elevated costs [10]. Nevertheless, as dementia progresses, individuals may require long-term care due to safety concerns, compromised quality of care, and heightened stress on informal caregivers associated with their increasing dependence and challenging neuropsychiatric symptoms. Indeed, for PwD residing in the community, the burdens of the disease largely fall upon informal caregivers [11]. Informal caregivers (hereafter also referred to as caregivers) are typically understood as individuals, whether family or others, who provide unpaid and ongoing assistance with basic or instrumental activities of daily living to someone with a disability or chronic illness [12]. The burden on informal caregivers is especially noticeable in in low- and middle-income countries [13], as well as in cultures where there exists a societal norm of family-based and intergenerational care, as observed in Mediterranean and Southern European countries, including Portugal [14, 15]. Therefore, informal caregivers typically play a crucial role in the decision-making process regarding the institutional placement of the PwD, with cultural factors often influencing this process.

Research on the institutionalization of PwD has revealed the multifaceted nature of the circumstances leading to their transition to long-term care. These encompass a range of factors, including sociodemographic, health-related, and psychological aspects pertaining to both PwD and their informal caregivers, as well as contextual variables.

Evidence synthesis studies indicate that certain sociodemographic characteristics of care recipients, such as advanced age, unmarried status, and living alone, predict institutional placement for PwD [4, 16, 17]. Additionally, caregivers’ sociodemographic traits, including higher levels of education, being employed, and higher income, were found to be associated with the institutionalization of PwD [4, 16]. On the other hand, conflicting findings have emerged regarding the dyads’ kinship, with being a spouse or a child of a PwD associated with either a higher or lower risk of institutionalization for the PwD [4, 16, 17].

Concerning disease-related factors, greater cognitive and functional impairment, the presence and severity of neuropsychiatric symptoms, and a diagnosis of Alzheimer’s disease are associated with heightened risks of institutional placement [4, 16–18].

Regarding caregivers’ psychosocial variables, the presence of caregiver burden, physical and mental health concerns, lower life satisfaction, and reduced health-related quality of life have been linked to an increased risk of institutionalizing the PwD [4, 16–18]. However, one review noted inconsistent findings regarding caregiver depression and physical health [18].

Finally, regarding the care context, studies inconsistently report associations between institutionalization and both the amount of hours dedicated to care and the use of community support services [4, 16–18].

In addition to these factors, the caregiver’s contemplation of future institutional care, though less researched than actual institutionalization, has gained recent attention [19–23]. Previous research has identified such contemplation as the single most important predictor of institutionalization for PwD [4, 24–27]. Caregivers often consider placing a relative with dementia in institutional care long before it becomes a reality, indicating that institutionalization unfolds gradually rather than as a sudden event [19]. This process, referred to as the ‘desire to institutionalize’, is often characterized by conflicted feelings, doubts, and guilt, which may persist even after the actual placement [20]. Current evidence indicates that the desire to institutionalize shares similar predictors with actual institutionalization, suggesting it may serve as a precursor [4, 19]. Studies have found that factors consistently associated with actual institutionalization, such as diminished autonomy, frequency and severity of neuropsychiatric symptoms in PwD, and caregiver burden, also present as predictors of the desire to institutionalize [20–22]. Research on the association between the desire to institutionalize and caregiver age, the social support received by caregivers, utilization of formal services by caregivers and care recipients, and the caregiver’s occupational status has produced inconsistent findings [20–22]. A few studies have made notable strides in addressing research gaps by examining a comprehensive set of modifiable factors, such as caregivers’ depression, burden, coping or perceived health [19, 22], as predictors of the desire to institutionalize. However, most studies on this topic have conducted recruitment in restricted contexts, such as at local or regional levels and through clinical settings (e.g., hospitals, memory clinics) [19–21, 23]. Moreover, most studies have relied on conventional statistical techniques, which, coupled with typically low sample sizes, may be limited in handling the number and complexity of variables associated with the desire to institutionalize. Hence, insights into the predictors of this desire remain somewhat restricted, as they are tied to the number of modifiable and non-modifiable variables simultaneously investigated, limited recruitment contexts, and the use of statistical techniques that may be insufficient in building a model to understand the complexity of these factors.

**Fig. 1:**
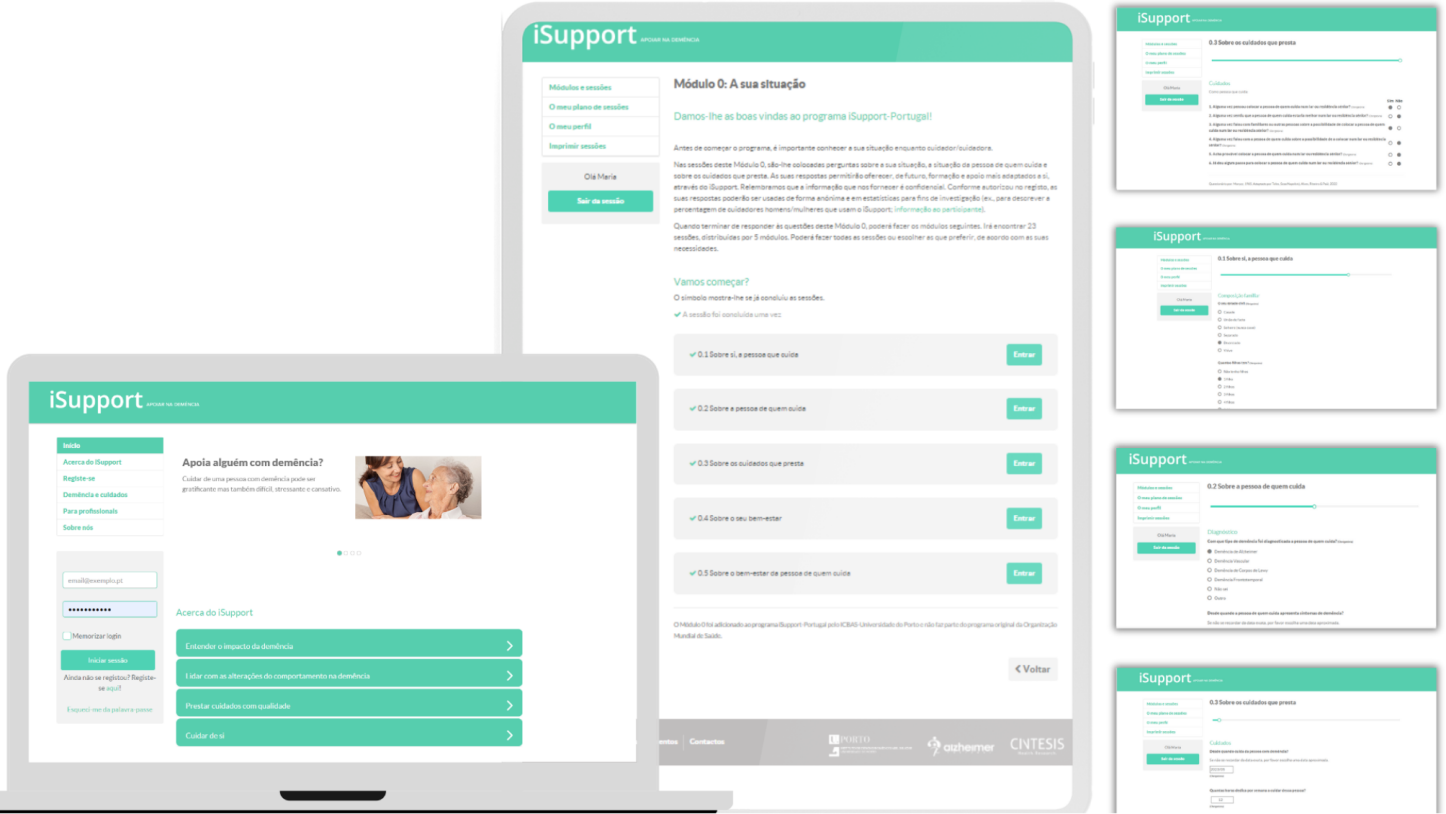
iSupport-Portugal: landing page and Module 0 screenshots

This study introduces a novel approach to researching dementia care dyads by utilizing an eHealth platform – iSupport-Portugal - to collect data remotely and nationwide. ‘iSupport’ is an online training and support program originally devised by the World Health Organization (WHO) to alleviate or prevent mental health issues commonly experienced by caregivers, while also facilitating their ability to maintain care at home [28]. It was developed as a self-managed program comprising five modules accessible around the clock. Akin to typical online interventions, the program is easily scalable at minimal marginal costs per additional user. iSupport has been or is currently undergoing adaptation in over 40 countries, apart from Portugal (e.g., Brazil [29], Greece [30], India [31], Indonesia [32], Japan [33], Switzerland [34], Spain [35]). iSupport-Portugal (see Fig. 1) stands as one of the pioneering culturally adapted versions, achieved through a multi-step methodological approach [36], and studied for its usability, acceptability [37], and feasibility [38]. Given the high rate of informal caregivers supporting a PwD in a recent national study (33%) [39], iSupport-Portugal is thought of as a helpful solution, particularly as new generations of caregivers assume caregiving roles.

iSupport-Portugal is currently evolving into a research-intervention platform with the capability to remotely collect data on dementia care dyads over time and nationwide, facilitating descriptive and predictive research. Web platforms and mobile apps are increasingly utilized as remote measurement tools (RMT), providing alternatives to traditional assessment methods and enabling real-time and longitudinal collection of health and behavioral variables in a cost-effective and non-intrusive manner [40]. iSupport-Portugal stands out as the first international iSupport platform that has been technically and scientifically enhanced to collect and export data on dementia care dyads. Before its public release, multiple preparatory steps were taken to ensure the integrity, security, and ethical handling of data collected through iSupport-Portugal. This encompassed optimizing data export processes and integrating a unique ‘diagnosis module’ into iSupport-Portugal, incorporating pre-selected assessment scales. The diagnosis module and other iSupport-Portugal forms enable the collection of sociodemographic, health, and psychosocial data from caregivers and PwD, covering numerous variables pertinent to research and conceptual frameworks concerning the desire for and institutionalization of the PwD (e.g., [4]). Following the initial iSupport usability and feasibility studies [37, 38], during which access was restricted to participants with a designated access code, iSupport became accessible to the public through free registration on isupport-portugal.pt.

This study investigates the desire to institutionalize a PwD in a sample of informal caregivers from Portugal, a country with a high prevalence of dementia and a strong reliance on informal care. It aims to examine the predictive value of a comprehensive range of sociodemographic, contextual, and psychosocial variables (modifiable and non-modifiable) in relation to the desire to institutionalize the PwD. Therefore, the study’s purpose is to develop a prediction model of caregivers’ desire to institutionalize a care recipient with dementia by mining user data from the eHealth platform isupport-portugal.pt. Data mining techniques can often overcome the shortcomings of traditional methods in investigating predictors and contribute with new discoveries [41]. Particularly for this study, it can add value in handling the numerous variables that are relevant to predicting the desire to institutionalize and uncovering hidden knowledge from the data.

## Materials and methods

### Study design

Observational, cross-sectional study resorting to primary data collected at baseline upon user registration on the eHealth platform isupport-portugal.pt, freely accessible to the public after registration.

### Participants

A sample of eligible individuals who completed registration on the isupport-portugal.pt platform between February 2023 and February 2024 was selected, making the sampling approach non-probabilistic. The platform’s inclusivity, catering to diverse user types such as health and social support professionals, necessitated filtering the sample to exclusively include caregivers. The study’s inclusion criteria encompassed users who consented to participate in research and were (i) aged 18 and over; (ii) residing in Portugal; (iii) providing unpaid care; (iv) to a community-dwelling person (not in long-term residential care); (v) diagnosed with dementia. In establishing eligibility criteria, no restrictions were applied concerning the time elapsed since the dementia diagnosis, the duration, or the number of weekly hours dedicated to caregiving, although these contextual variables were collected (see Variables and measures). This approach offered more variability in searching for the emergence of patterns from the data by applying data mining techniques (see Data analysis). After the public release of iSupport-Portugal, it was disseminated through multiple channels, including professional referrals, media, and institutional websites. This approach allowed users to become acquainted with the platform and, consequently, be recruited through various of those channels.

Upon registration at isupport-portugal.pt, users who declared themselves to be informal caregivers were provided with full information and invited to participate in research. Informed consent was obtained online through the user’s personal account and participants were invited to fill in post-registration questionnaires through their user area. Caregivers who choose not to participate in the study were not hindered from using the program in any way. This study received a positive opinion from the Ethics Committee for Health of the Faculty of Medicine of the University of Porto (ref: 76/CEFMUP/2022). Additionally, the Data Protection Officer of the University of Porto conducted an evaluation of data protection requirements for isupport-portugal.pt.

### Variables and measures

The study data were exclusively collected online during registration at isupport-portugal.pt and through fill-in forms within the user’s personal area. Upon completion of the registration form for iSupport-Portugal and acceptance to participate in the research, caregivers provided basic sociodemographic information concerning both themselves and the PwD. A “diagnosis module” (Module 0) was incorporated into the platform to gather context-of-care, clinical, and psychosocial variables pertaining to the caregiver-PwD dyads. This module precedes the five intervention modules comprising iSupport (see Introduction). All data concerning the PwD were obtained through the caregivers’ reports.

#### Sociodemographic data on caregivers and the PwD

Informal caregivers were requested to furnish sociodemographic information about themselves and the person they were caring for. This included details such as age, gender, years of schooling, and marital status for both parties. Additionally, caregivers provided information about their occupational and parental status, including the number of children and those in cohabitation. The living arrangements of the PwD were also examined, as per the eligibility criteria outlined in the Participants section. Reporting that the PwD resided in institutional care resulted in the exclusion of the participant.

#### Context-of-care and service use data

Context-of-care data gathered from caregivers included the kinship (or other relationship) and cohabitation with the PwD, duration of caregiving, weekly caregiving hours, access to regular caregiving support, and the type of support received (unpaid, paid and specialized, paid but unspecialized). Additionally, caregivers indicated, from a predefined list, the services utilized by either the PwD (including home care services, home health services, day or night centers, cognitive or occupational therapy, Memory Cafés) or themselves (including psychoeducational, support or mutual aid groups, mental health counselling, and Memory Cafés) at the time of response.

#### Clinical profile and functionality data on the PwD

Caregivers provided information on the diagnosis of the PwD, including details about the disease-causing dementia (if known) and the time elapsed since the medical diagnosis. They assessed the functional independence of the PwD subjectively using a single item and by completing the Barthel Index [42], European-Portuguese version [43]. The Barthel Index is a widely used 10-item instrument for evaluating functional independence in activities of daily living. Higher total scores (ranging from 0 to 20) indicate greater independence, with proposed cut-offs for total dependence (0–8 points), severe dependence (9–12 points), moderate dependence (13–19 points), and independence (20 points).

Neuropsychiatric symptoms of the PwD, recognized as core features of Alzheimer’s disease and other dementias, and significant contributors to caregiver psychological distress [44] and institutionalization [45], were assessed using the Neuropsychiatric Inventory Questionnaire (NPI-Q) [46], European-Portuguese version [47]. This instrument evaluates: (i) the presence or absence of 12 neuropsychiatric symptom domains (delusions, hallucinations, agitation/aggression, dysphoria/depression, anxiety, euphoria/elation, apathy/indifference, disinhibition, irritability/lability, aberrant motor behaviors, nighttime behavioral disturbances, and appetite/eating disturbances); (ii) the severity of reported symptoms in the past month (mild, moderate, or severe), with total severity scores ranging from 0 to 36; and (iii) caregiver distress for each reported symptom, rated on a 6-point scale, with total distress scores ranging from 0 to 60 points, where higher scores indicate greater caregiver distress.

#### Psychosocial data on informal caregivers

Caregivers completed several psychosocial measures: (i) the Zarit Burden Interview, a well-established 22-item instrument assessing perceived burden. Total scores range from 0 to 88 points, with higher scores indicating greater burden [48, 49]; (ii) anxiety and depression symptoms were assessed using the respective subscales of the Hospital Anxiety and Depression Scale (HADS), a 14-item instrument (7 items for depression and 7 for anxiety). Total scores per subscale range from 0 to 21, with higher scores indicating more severe symptoms. Cut-off scores for a borderline case are 8–10 points, and scores ≥ 11 suggest a clinical case [50, 51]; (iii) quality of life was measured with the WHOQOL-BREF [52, 53], a 26-item instrument covering four quality of life domains - physical, psychological, social relationships, and environment - as well as items relating to overall quality of life, with higher total scores indicating higher quality of life; (iv) positive feelings resulting from caregiving were assessed with the Positive Aspects of Caregiving (PAC), an 11-item instrument with total scores ranging from 11 to 55, with higher scores indicating more positive perceptions of caregiving [54, 55]; and (v) coping orientation to problems experienced regarding caregiving was measured with Brief-COPE, a 28-item instrument where each item is scored from 0 to 3 in the Portuguese version. This tool can determine primary coping styles - problem-focused, emotion-focused, and avoidant – as well as 14 coping facets [56, 57].

#### Caregivers’ desire to institutionalize

The caregiver’s inclination to consider the institutionalization of the PWD was gauged using the Desire to Institutionalize Scale (DIS) [25]. Previous studies consistently show that DIS has a strong predictive ability for future institutionalization [19, 58, 59]. DIS was translated into European Portuguese and evaluated for its psychometric properties, revealing good structural validity, high internal consistency (α = 0.802), and association with caregiver, care recipient, and contextual variables known to influence institutional placement [60]. The instrument comprises six items, each answered with a yes or no response, yielding total scores ranging from 0 to 6 points. These items address various stages of contemplation for institutional placement, spanning from mere consideration to taking active steps. Given the negatively skewed distribution of responses on the DIS (Median (Mdn) = 2), and to enhance model interpretability, caregivers were divided into two groups: those scoring 0 on all items (DIS total score = 0) were classified into the ‘no desire to institutionalize’ group (no DI), while those endorsing at least one item on the DIS (DIS total score ≥ 1) were placed in the ‘desire to institutionalize’ group (DI). Previous research has treated the DIS as a dichotomous variable [19, 22, 59].

### Data analysis

Descriptive statistics were computed for all variables, encompassing measures of central tendency (mean, median) and dispersion (standard deviation, first and third quartile) for continuous variables, as well as frequencies and percentages for categorical variables.

The selected model was a classification tree. This method was chosen due to its non-parametric nature, enabling the modelling of nonlinear relationships and interactions between variables without imposing prior assumptions about the data distribution. The classification tree was tuned and pruned to prevent overfitting, with specified parameters including a minimum of 10 cases for a split and at least 7 cases in each bucket. A maximum tree depth of 20 was set. However, in this case, the maximum depth was never reached since the rule regarding the minimum cases per bucket was triggered first. Rules were extracted from the final tree, and support and confidence were calculated [61]. Additionally, variable importance was extracted from the tree, showing the relative influence of each variable in the classification process [62]. The model’s performance was assessed using the following metrics: accuracy, sensitivity, specificity, positive predictive value (PPV), and negative predictive value (NPV). For each metric, 95% confidence intervals (CIs) were computed using bootstrapping with 5000 repetitions [63]. Bootstrapping is a statistical technique that entails random sampling from the original dataset with replacement numerous times to create multiple bootstrap samples to estimate the distribution of a statistic. This method is particularly useful when the underlying data distribution is unknown or when dealing with small sample sizes, as it allows for the estimation of the sampling distribution of a statistic without making stringent assumptions about the population distribution. All analyses were performed using R version 4.2.2 (2022-10-31) and RStudio 2023.06.0 Build 421, utilizing the packages “rpart” for classification trees, variable importance, and model training, as well as “boot” for confidence interval estimation.

## Results

### Characterization of study participants

After excluding observations with missing values for the DIS (*n* = 26, 20.0%), this study included a sample of 104 eligible informal caregivers of PwD. Table 1 presents descriptive statistics on socio-demographic, context of care, clinical, and psychosocial variables for all participants and categorizes them into groups based on their desire – either with desire (DI) or without desire (no DI) – to institutionalize the care recipient with dementia.

The average age of informal caregivers was 52.5 years (range: 21 to 83 years), with the majority being female (82.5%). A significant portion were employed (65.4%) and, on average, had a high level of education (Mean (M) 15 years of schooling). Most caregivers were either children or grandchildren of the PwD (70.2%). Among both offspring and spousal caregivers, a majority had children (57.5% and 89.5%, respectively), and most offspring caregivers lived with their children (73.8%), suggesting they were likely balancing care responsibilities for a parent alongside caring for their own children.

Over half of the caregivers resided with the person they were caring for (57.7%), and the majority provided intensive care (Median (Mdn) 24 h per week, range: 1–168 h) over a long duration (Mdn 2.8 years), often supplemented with regular formal or informal support (69.2%). The median caregiving duration was lower in the group showing a desire to institutionalize (Mdn 2.8 vs. 3.2). Overall, although more than half of the care recipients utilized community services (57.7%), only about a third of caregivers sought services for their own benefit (34.6%).

The persons with dementia in their care had an average age of 78.5 years, ranging from 45 to 93 years old, indicating that cases of young onset dementia are represented in the study sample. On average, the care recipients are younger in the group showing a desire to institutionalize (M 77.3 vs. 80.6). The majority of PwD were female (69.2%), had low levels of education (Mdn 4 years of schooling), and over half were married or in a union (58.7%). The median years of schooling of the PwD is lower in the group showing a desire to institutionalize (Mdn 4 vs. 9).

In terms of their clinical profile, Alzheimer’s disease was the most frequently reported diagnosis (47.6% of cases). Regardless of the specific dementia diagnosis, the median time since diagnosis was 3 years (range: <1–15.8 years). For over a third of caregivers, their care recipients were perceived as moderately dependent, as indicated by scores for functional independence on the Barthel Index (Mdn 14, range: 0–20). According to caregivers’ assessments, 93.3% of PwD exhibited at least one neuropsychiatric symptom (Mdn 5, range: 0–12), with a median severity score of 9.5 for positive symptoms. Despite the high prevalence of neuropsychiatric symptoms, caregivers’ distress caused by them was relatively low (Mdn 11).

On average, informal caregivers reported high levels of perceived burden (M 36.1), with a cutoff of 21 commonly recognized as a threshold for indicating burden [48]. Caregivers in the desire to institutionalize group score on average higher (M 39.5) for burden than those in the group with no desire to institutionalize (M 29.8).

However, overall, caregivers in this sample also expressed a relatively high recognition of positive aspects of caregiving (M 33.8 on the PAC scale; range 11–55). On average, caregivers scored above the cutoff score (≥ 8 points) for clinically relevant anxiety symptomatology (M 9.1 points on HADS-A) and below the cutoff for clinically relevant depression symptomatology (M 7.5 points on HADS-D). Overall, 67.7% and 47.0% of caregivers would be classified as borderline or abnormal cases for anxiety and depression, respectively. On average, caregivers in the group showing a desire to institutionalize scored higher in anxiety (M 9.7 vs. 8.0).

When evaluating their quality of life, participants rated the social relationships domain the lowest (M 55.6, SD 21.3) on the WHOQOL-BREF [52, 53] (transformed scores, 0 to 100), in comparison to the physical (M 68.8, SD 18.3), psychological (M 64.4, SD 16.2), and environmental domains (M 63.9, SD 17.0), as well as their general quality of life (M 60.4, SD 19.3).

**Table 1:** Descriptive statistics for all participants, caregivers with desire (DI) and no desire (no DI) to institutionalize

Variable	Total *N*	Total Descriptive statistics	DI *N*	DI Descriptive statistics	No DI *N*	No DI Descriptive statistics
**Socio-demographic**						
***Informal caregiver***						
Age (years), M (SD)	104	52.5 (12.9)	69	51.6 (12.3)	35	54.3 (14.0)
Gender, Female, n (%)	104	85 (82.5)	69	59 (85.5)	35	26.0 (74.3)
Years of schooling, M (SD)	103	15.0 (4.6)	69	14.9 (4.4)	34	15.1 (4.9)
Occupational status, Employed, n (%)	104	68 (65.4)	69	51 (73.9)	35	17 (48.6)
Marital status, Partnered ^†^, n (%)	104	61 (58.7)	69	45 (65.2)	35	16 (45.7)
Children, Yes						
Among all caregivers, n (%)	104	64 (61.5)	69	45 (65.1)	35	19 (54.3)
Among offspring caregivers, n (%)	73	42 (57.5)	48	31 (64.6)	25	11 (44.0)
Among spousal caregivers, n (%)	19	17 (89.5)	13	11 (84.6)	6	6 (100)
Cohabiting children, Yes						
Among all caregivers with children, n (%)	64	38 (59.4)	45	29 (64.4)	19	9 (47.7)
Among offspring caregivers with children, n (%)	42	31 (73.8)	31	24 (77.4)​​	11	7 (63.6)
Among spousal caregivers with children, n (%)	17	3 (17.6)	11	3 (27.3)	6	0 (0)
***Person with dementia (PwD)***						
Age (years), M (SD)	104	78.5 (8.2)	69	77.3 (8.2)	35	80.6 (7.9)
Gender, Female, n (%)	104	72 (69.2)	69	47 (68.1)	35	25 (71.4)
Years of schooling, Mdn (Q1, Q3)	103	4.0 (4.0, 10.0)	68	4.0 (4.0, 9.0)	35	9.0 (4.0, 15.0)
Marital status, Partnered ^†^, n (%)	104	61 (58.7)	69	39 (56.5)	35	22 (62.9)
**Context-of-care & service use**						
Kinship with the PwD	104		69		35	
Offspring, n (%)		73 (70.2)		48 (69.6)		25 (71.4)
Spouses, n (%)		19 (18.3)		13 (18.8)		6 (17.1)
Other, n (%)		12 (11.5)		8 (11.6)		4 (11.4)
Cohabitation with the PwD, Yes, n (%)	104	60 (57.7)	69	34 (49.3)	35	26 (74.3)
Caregiving duration (years), Mdn (Q1, Q3)	101	2.8 (1.2, 5.7)	68	2.8 (1.0, 5.7)	33	3.2 (1.2, 5.9)
Hours caring (per week), Mdn (Q1, Q3)	104	24.0 (10.0, 50.0)	69	24.0 (11.0, 48.0)	35	24.0 (10.0, 64.0)
Support for caregiving, Yes, n (%)	104	72 (69.2)	69	47 (68.1)	35	25 (71.4)
PwD service use, any service ^#^, Yes, n (%)	104	60 (57.7)	69	42 (60.9)	35	18 (51.4)
Caregiver service use, any service ^#^, Yes, n (%)	104	36 (34.6)	69	23 (33.3)	35	13 (37.1)
**Clinical profile and functionality: PwD**						
Type of dementia	103		68		35	
Alzheimer’s disease, n (%)		49 (47.6)		33 (48.5)		16 (45.7)
Vascular dementia, n (%)		17 (16.5)		9 (13.2)		8 (22.9)
Frontotemporal dementia, n (%)		14 (13.6)		10 (14.7)		4 (11.4)
Dementia with Lewy bodies, n (%)		8 (7.8)		5 (7.4)		3 (8.6)
Other/unknown, n (%)		15 (14.6)		11 (16.1)		4 (11.5)
Time elapsed since diagnosis (years), Mdn (Q1, Q3)	102	3.0 (0.8, 5.7)	68	2.8 (1.1, 5.7)		1.3 (0.8, 1.5)
Dependence level	104		69		35	
Mild, n (%)		16 (15.4)		13 (18.8)		3 (8.6)
Moderate, n (%)		36 (34.6)		23 (33.3)		13 (37.1)
Severe, n (%)		26 (25.0)		17 (24.6)		9 (25.7)
Total, n (%)		26 (25.0)		16 (23.2)		10 (28.6)
Functional independence ^a^, Mdn (Q1, Q3)	89	14.0 (6.0, 18.0)	60	14.0 (6.0, 19.0)	29	15.0 (9.0, 18.0)
Neuropsychiatric symptoms ^b^	90		60		30	
Severity, Mdn (Q1, Q3)		9.5 (5.0, 14.0)		10.0 (6.0, 14.0)		8.0 (3.0, 12.0)
Present symptoms, Mdn (Q1, Q3)		5.0 (3.0, 7.0)		5.0 (3.0, 7.0)		4.0 (1.0, 7.0)
**Psychosocial: caregiver**						
Perceived burden ^c^, M (SD)	103	36.2 (13.6)	68	39.5 (12.6)	35	29.8 (13.3)
Distress, PwD neuropsychiatric symptoms, Mdn (Q1, Q3) ^d^	90	11.0 (5.0, 18.0)	60	11.0 (6.0, 19.0)	30	11.0 (3.0, 17.0)
Anxiety symptoms ^e^, M (SD)	99	9.1 (3.3)	64	9.7 (3.3)	35	8.0 (3.0)
Depression symptoms ^e^, M (SD)	100	7.5 (4.2)	65	7.5 (4.1)	35	7.5 (4.4)
Quality of life ^f^						
General, M (SD)	100	6.8 (1.5)	65	6.8 (1.6)	35	7.0 (1.4)
Physical, M (SD)	99	25.7 (5.1)	64	25 (4.8)	35	26.9 (5.5)
Psychological, M (SD)	99	21.5 (3.9)	64	21.1 (3.8)	35	22.1 (4.0)
Social relationships, M (SD)	99	9.7 (2.6)	64	9.7 (2.6)	35	9.7 (2.6)
Environment, M (SD)	99	28.4 (5.4)	64	28.2 (5.2)	35	28.9 (5.9)
Positive aspects of caregiving ^g,^, M (SD)	104	33.8 (9.8)	69	32.6 (10.5)	35	36.1 (7.9)
Coping styles ^h^						
Problem-Focused Coping, M* (SD)	95	1.4 (0.6)	63	1.4 (0.6)	32	1.3 (0.6)
Emotion-Focused Coping, M* (SD)	94	1.1 (0.4)	62	1.1 (0.4)	32	1.0 (0.4)
Avoidant Coping, M* (SD)	96	0.6 (0.4)	63	0.6 (0.4)	33	0.5 (0.4)

*Abbreviations*: N/n – number of participants; M – mean; Mdn – median; SD - standard deviation; IQR- interquartile range; DI - desire to institutionalize (Desire to Institutionalize Scale ≥ 1); No DI - no desire to institutionalize (Desire to Institutionalize Scale = 0)

*Notes*: ^†^ Includes married or in a de facto union; ^#^ Includes the services described in the ‘Materials and Methods section’; * Presents a grand mean/pooled mean; ^a^ Measured with Barthel Index, total scores can range from 0 to 20 points; ^b^ Measured with the Neuropsychiatric Inventory Questionnaire (NPI-Q), total severity score can range from 0 to 36; ^c^ Measured with the Zarit Burden Interview – 22 items, total scores can range from 0 to 88 points; ^d^ Measured with the NPI-Q, total NPI-Q distress scores can range from 0 to 60 points; ^e^ Measured with the Hospital Anxiety and Depression Scale, respective subscales for anxiety and depression, cutoff for borderline or abnormal ≥ 8 points, total scores per subscale can range from 0 to 21 points; ^f^ Measured with the WHOQOL-BREF - each item can range from 1 to 5 points, reports on raw scores for each QoL domain; ^g^ Measured with the Positive Aspects of Caregiving scale (PAC), total scores can range from 11 to 55 points; ^h^ Measured with the Coping Orientation to Problems Experienced Inventory (Brief-COPE), the average score per coping style can range from 0 to 3 points in the Portuguese version

### Desire to institutionalize

Roughly two-thirds of caregivers (*n* = 69, 66.3%) have endorsed at least one item on the DIS, thereby categorizing them into the “desire to institutionalize” group. The remaining caregivers (*n* = 35, 33.7%) scored 0 on the DIS, indicating no inclination to institutionalize a PwD under their care. An overall median score of 2 (Q1 0, Q3 3) was obtained. Analysis of the distribution of total scores on the DIS suggests a mild inclination towards institutionalization, with most caregivers in the “desire to institutionalize” group (*n* = 69) endorsing 3 or fewer items (*n* = 44, 63.8%), compared to those endorsing more than 3, up to all 6 items (*n* = 25, 36.2%).

As a caregiver, 41.4% (*n* = 43) of participants reported having considered a nursing home for the PwD in their care, but only 18.3% (*n* = 19) have ever felt that the care recipient would be better off in a nursing home. While 51.9% (*n* = 54) have discussed the institutionalization of the PwD with family or others, the majority have never done so with the PwD (77.9%, *n* = 81). Although only 23.1% (*n* = 24) have taken steps towards institutional placement, 40.4% (*n* = 42) consider institutionalization to be a likely outcome for their care recipient.

### Classification tree

**Fig. 2:**
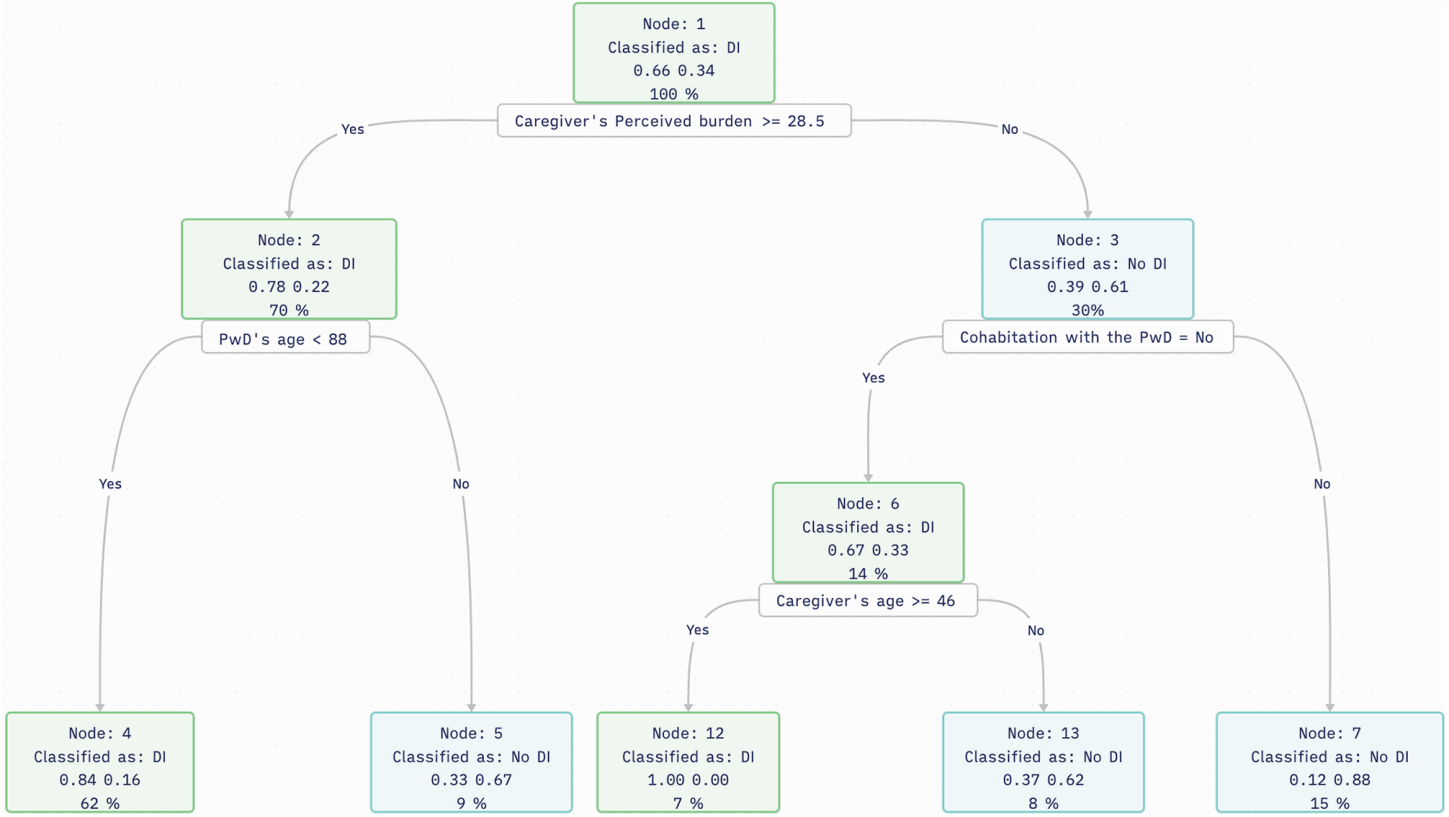
Plot of the final pruned tree for classifying caregivers with the desire to institutionalize. *Abbreviations*: PwD - person with dementia; DI – Desire to institutionalize; No DI – no desire to institutionalize; *Notes*: caregiver’s perceived burden was measured with the Zarit Burden Interview (ZBI-22)

Figure 2 depicts a graphical representation of the decision model utilized in the analysis. The nodes represent decision points, while the edges indicate the directional flow of the decision process. The percentages at each node reflect the probability of the corresponding outcome e.g. in node 2 one can find 70% of all the cases of which 78% fall into the DI group and 22% fall into the no DI group; since most of the cases fall into the first, this node is classified as DI (identified with a 1 on top of the node). This offers a quantified insight into the decision-making process. The selected root was the variable “Caregiver’s perceived burden”, and the split point 28.5.

### Variable importance extracted from the prediction model

**Fig. 3:**
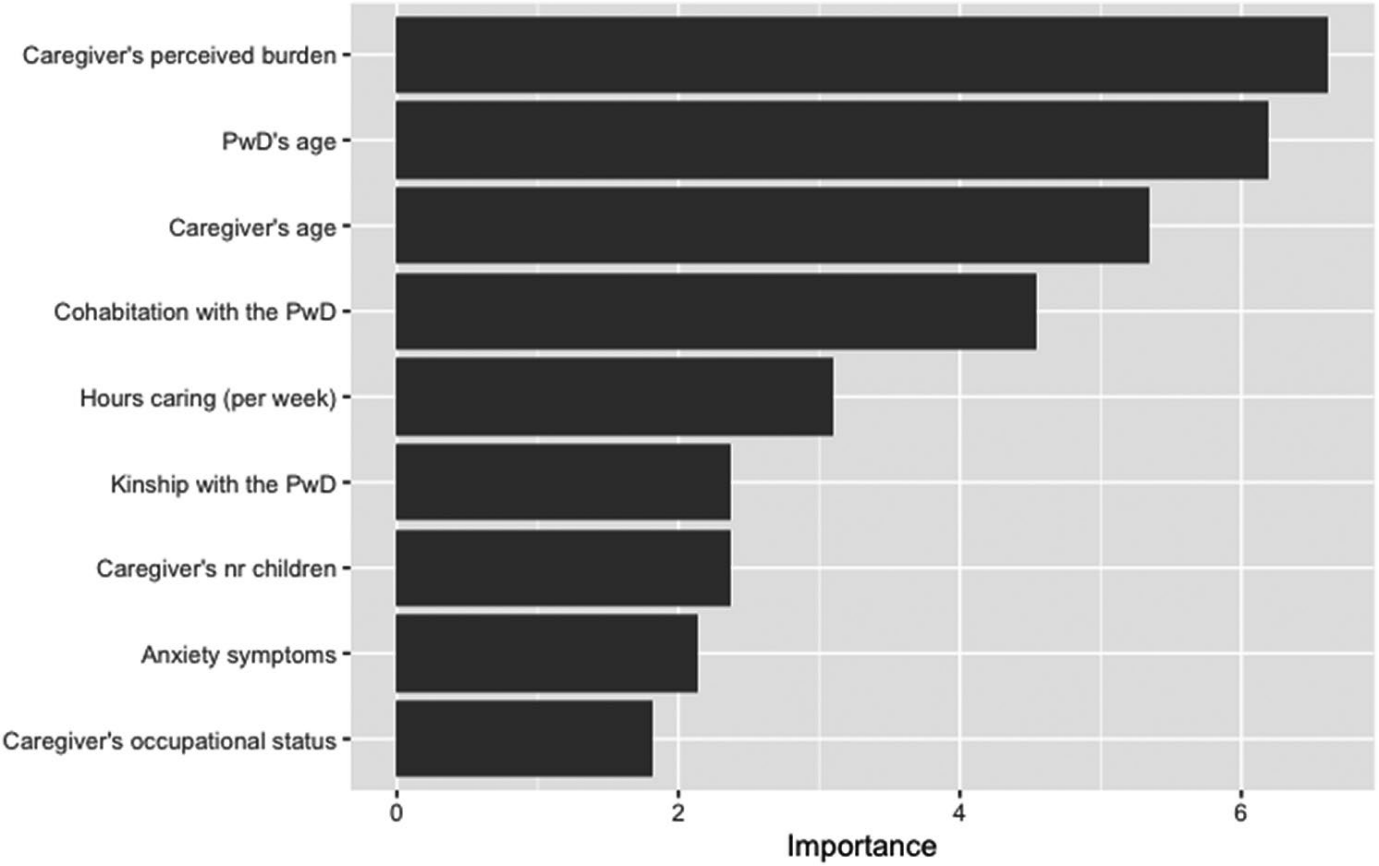
Variable importance calculated from the tree for classifying caregivers with the desire to institutionalize. *Abbreviations*: PwD - person with dementia; *Notes*: caregiver’s perceived burden was measured with the Zarit Burden Interview (ZBI-22); anxiety symptoms were measured with the Hospital Anxiety and Depression Scale (HADS-A); see Table 1 for categories comprising nominal variables

Figure 3 provides another perspective of the model. Given that some variables exhibit strong associations, the decision between one variable over another may hinge on just a few cases, potentially hiding the importance of one variable that might not appear in the final model. Furthermore, some variables might appear multiple times in the tree, further from the tree’s root, thereby also mitigating the perceived importance. A higher variable importance value indicates that a variable is more crucial for predicting the outcome because it has contributed more to reducing uncertainty or impurity across the splits in which it was used. Variables with low or zero importance values have little to no effect on the outcome prediction within the context of the constructed tree. The actual numerical value of variable importance (displayed on the x-axis of Fig. 3) indicates the relative contribution of that variable compared to others in the model and depends on the complexity of the model. Hence, Fig. 3 displays a measure of association between the variables and the outcome (desire to institutionalize), even for variables not chosen for the final pruned model. Caregiver’s perceived burden, PwD’s age, caregiver’s age, and cohabitation with the PwD were the primary contributing variables in the model, all of which are included in the final model depicted by the classification tree (see Fig. 2).

### Analysis of extracted rules

From the model, rules were extracted and are presented in Table 2. Three rules stand out for their *confidence*: rules 1, 3 and 5. Rule number 1, where the caregiver’s perceived burden is equal to or greater than 28.5 (given by the algorithm) based on scores from the Zarit burden Interview and the PwD is younger than 88, exhibits very high confidence and accounts for the majority of cases. In contrast, rule number 3, where the caregiver has scored less than 28.5 on perceived burden, is aged 46 or over, and does not cohabit with the PwD, despite its high confidence, only accounts for 7 cases. Among these cases, while the variable of cohabitation rated higher in importance than kinship for predicting the outcome (see Fig. 3), it’s notable that for couples, cohabitation is implied. Therefore, it’s worth noting that the majority of cases in this group (6 cases) involve children of the PwD, with one representing another relationship. Rule number 5, where the caregiver scores below 28.5 on perceived burden and cohabits with the PwD, also shows high confidence and is the only one of these three that favors the class that does not desire institutionalization.

**Table 2:** Rules extracted from the tree for classifying caregivers with the desire to institutionalize

Rule nr.	Rule description	Outcome	Support	Confidence
1	Caregiver’s perceived burden > = 28.5 & PwD’s age < 88	DI	64	0.83
2	Caregiver’s perceived burden > = 28.5 & PwD’s age > = 88	No DI	9	0.64
3	Caregiver’s perceived burden < 28.5 & Cohabitation with the PwD = No & Caregiver’s age > = 46	DI	7	0.89
4	Caregiver’s perceived burden < 28.5 & Cohabitation with the PwD = No & Caregiver’s age < 46	No DI	8	0.60
5	Caregiver’s perceived burden < 28.5 & Cohabitation with the PwD = Yes	No DI	16	0.83

*Abbreviations*: PwD - person with dementia; DI - Desire to institutionalize

*Notes*: Caregiver’s perceived burden was measured with the Zarit Burden Interview − 22 items (ZBI-22); *support* corresponds to the number of observations covered by the rule; *confidence* corresponds to the prediction accuracy for each class

### Performance metrics of the model

The model demonstrates overall good predictive performance. Table 3 presents the confusion matrix. The model achieves an accuracy of 0.83, with a 95% confidence interval (CI) ranging from 0.75 to 0.89. The sensitivity of the model is 0.88, with a 95% CI from 0.81 to 0.95, while the specificity is 0.71, with a 95% CI from 0.56 to 0.86. The positive predictive value (PPV) is recorded at 0.86, with a 95% CI from 0.77 to 0.93, and the negative predictive value (NPV) is 0.76, with a 95% CI from 0.61 to 0.90. It’s noteworthy to mention the model’s commendable accuracy and its higher sensitivity compared to its specificity, indicating that the model is better at predicting caregivers with the desire to institutionalize than at predicting caregivers without such a desire.

**Table 3:** Confusion matrix of the model’s predictions, regarding caregivers’ desire to institutionalize

	DI	No DI
Predicted DI	61	10
Predicted No DI	8	25

*Abbreviations*: PwD - person with dementia; DI - Desire to institutionalize

## Discussion

### Main findings and contributions

This study focused on describing and developing a predictive model for informal caregivers’ desire to institutionalize a PwD by analyzing data gathered from an eHealth platform designed to support these caregivers - iSupport-Portugal. Classification tree modelling was employed, enabling the visualization of a set of rules for predictions. The resulting model, anchored in caregivers’ perceived stress as the root of the classification tree and incorporating caregivers’ age, PwD’s age, and cohabitation status, demonstrated strong predictive performance overall, achieving high accuracy (0.83) and particularly commendable sensitivity (0.88, compared to a specificity of 0.71). These findings suggest that the model is particularly useful in identifying caregivers with actual desire to institutionalize their care recipients.

This research distinguishes itself from previous studies on the topic in several key aspects. Firstly, it innovates in data collection methods by employing a remote measurement tool (iSupport-Portugal) to gather nationwide data, thus overcoming the limitations of restricted recruitment contexts commonly observed in the literature (see Introduction). Secondly, it stands out in the number and diversity of predictors considered in deriving the model, encompassing a comprehensive array of potentially influential sociodemographic, contextual, and psychosocial variables, both modifiable and non-modifiable. Thirdly, the study adopts a classification tree approach, offering a simplified model derived from the multifaceted and complex factors influencing the desire to institutionalize a PwD, thus enhancing the model’s interpretability and potential, as well as its ease of applicability.

As for the innovation in data collection methods, iSupport-Portugal stands out as the first internationally tailored iSupport platform utilized for descriptive and predictive research on dementia care dyads. This approach offers a blueprint that could be replicated by other iSupport country-specific platforms or similar eHealth interventions. The primary advantage lies in its ability to gather cost-effective and territorially diverse data. In contrast, studies relying on conventional data collection methods have predominantly been confined to recruitment in clinical settings and at local or regional levels [19–21, 23]. However, the gains in cost-effectiveness are not without trade-offs and limitations, particularly concerning the characteristics of participants recruited through this method (see Limitations and future research).

The study also provides a cultural perspective on the topic of the desire to institutionalize, conducted in a country with a high prevalence of dementia and a prevailing reliance, as well as social pressure, on families to care for dependent individuals, characteristic of Mediterranean and Southern European countries [64]. In Southern European countries, reflecting a ‘familialistic’ approach to welfare, the needs of older persons tend to be more frequently addressed by informal caregivers, mainly women [65, 66]. Co-residential care, which is typically a more intense form of care, is also more frequent in the Southern European region [65]. Despite one could expect, accounting for this context, a low expression of the desire to institutionalize, 66.3% of caregivers in this sample endorsed at least one item on the DIS, a figure similar to or higher than what was found in previous studies (e.g., 63.4% in [22], Belgium; 50% in [19], Ireland). Additionally, in line with prior research (e.g., [19]), this study suggests that caregivers may begin considering the future institutional placement of the care recipient relatively early in the caregiving journey, with participants in the DI group providing care for a median of 2.8 years. These trends may be related to the significant presence of children of the PwD in this sample (70%), as acculturation and globalization processes may lead to the restructuring of pre-existing configurations of informal care, with alternative caregiving arrangements such as institutional placement or paid care at home becoming more common [67]. Among Southern European countries, previous research has underscored differences in the development of services and support for older adults and informal caregivers in response to demographic and social changes [68]. Portugal stood out compared to other Southern European countries (e.g., Greece), due to higher levels of women’s participation in the labor market, leading to increased demands for formal support services [68]. Yet, cultural factors shape motivations and willingness to provide informal care and influence caregiving expectations, including definitions of what constitutes ‘good’ and ‘bad’ care [67]. Indeed, although 40.4% of caregivers consider institutionalization likely and 51.9% have discussed it with family or others, only 18.3% believe that the PwD would be better off in a nursing home, indicating the presence of conflicting feelings and moral distress that may affect the caregiver’s perceived burden.

As for the number and variety of predictors considered to derive the model, it is noteworthy that some previous studies have made remarkable progress, particularly in the inclusion of modifiable and often overlooked variables concerning the desire to institutionalize. These variables may encompass coping styles, caregiver’s employment status, or access to support services (e.g., [19, 22]). This study has also evaluated such variables while incorporating others that have been overlooked in this field of research, such as the number of children and the number of children in cohabitation, particularly among caregivers supporting a parent. Indeed, considering that the iSupport-Portugal user base comprises a high number of offspring caregivers (above 70%), determining whether being part of the so-called “sandwich” generation would influence the desire to institutionalize was deemed relevant. Previous evidence has indicated that those who are coordinating care for both a parent and children provide as intensive care as non-sandwich caregivers, have higher participation in the labor force, and experience more caregiving-related overload [69]. Although the number of children was not included in the final model, it ranked among the most important variables for predicting the outcome (see Fig. 3). It ranked above other variables related to the clinical status of the PwD, such as functional independence or neuropsychiatric symptoms. Neuropsychiatric symptoms, previously identified as predictors of institutional placement [45], may have been overshadowed by the fact that they are also a primary contributor to caregiver burden [44]. In the context of the prediction tree, caregiver’s perceived burden emerges as the most important variable for predicting the outcome.

Overall, as evidenced by the ranking of variable importance (Fig. 3), this study aligns with previous research demonstrating that sociodemographic variables such as the caregiver’s and care recipients’ age, cohabitation, and caregiver’s occupational status are influential of the caregiver’s desire to institutionalize [19–22]. Interestingly, cohabitation ranks higher than kinship with the PwD in the variable importance, consistent with prior research suggesting that sharing the same household, rather than the type of relationship within the dyads, is more influential on the desire to institutionalize and on actual institutionalization [22, 27, 70]. Moreover, in line with previous research indicating perceived burden as the most significant factor influencing the desire to institutionalize [19, 22, 59], it is unsurprising that this variable was selected by the model as the tree root, and thus deemed the most crucial for predicting the outcome. On average, caregivers in this study reported high burden (M = 36.1), consistent with previous national research findings (e.g., M 36.2 in [55]).

Indeed, in terms of achieving a simplified and highly interpretable model from this study, the variables rated as most important for predicting the outcome led to a set of only five simple rules within the classification tree framework. For the tree root – the caregivers’ perceived burden – the split point was derived at a score of 28.5 on the Zarit Burden Interview (ZBI, 22 items). Traditionally, ZBI scores of 21 to 40 have been indicative of mild to moderate burden [48]. However, for the Portuguese context, cut-offs have not yet been properly culturally or clinically validated, and recommendations are to use total scores [71]. Departing from the caregiver’s perceived burden, these rules establish that having a ZBI-22 score of 28.5 and above, along with caring for someone aged less than 88 years, results in a desire to institutionalize the PwD (rule 1, confidence 0.83), while scoring the same and caring for someone aged 88 or over would lead to no desire to institutionalize (rule 2, confidence 0.64). This apparently counterintuitive result may be explained by caregivers’ expectations regarding the anticipated duration of care and their perceived ability to sustain caregiving for that duration, intersecting with the concept of perseverance time [72]. Despite experiencing burden, caregivers of older PwD may choose to defer their own needs and endure care demands in the expectation that care will be provided for a shorter timeframe. Conversely, caregivers facing burden while caring for younger individuals may be more willing to institutionalize due to the expectation of having to endure caregiving for a longer period.

Regarding caregivers experiencing less stress (ZBI < 28.5, rules 3, 4 and 5), both the cohabitation with the PwD and the caregiver’s age are shown to be influential. Caregivers with lower perceived burden who live with the person in care are classified as having no desire to institutionalize (rule 5, 0.83 confidence). Previous research has suggested that cohabitation, more than the kinship between the dyads, protects against the desire to institutionalize, most likely due to the more intense relationship between cohabitating dyads, as well as the impact of separation by institutionalization on both the caregiver and the care recipients [22]. Moreover, non-spousal caregivers who do not live with the PwD are more likely to face competing demands, such as holding a paid job and assisting their children [22]. In rules 3 and 4, caregivers with lower burden who are not cohabitating with the PwD, are differentiated according to their age being equal to/higher (rule 3, outcome: DI) or lower (rule 4, outcome: No DI) than 46. With respect to caregiver’s age, previous studies have been inconsistent, either showing an association of the desire to institutionalize with being younger (e.g., [21]), or older (e.g., [22]). In this study, rule 3 applies to caregivers who are older and, as presumed by the non-cohabitation condition, do not include spousal caregivers, mostly being children of the PwD.

The rules derived for the model are not only simple, as they involve only four variables, three of which are basic sociodemographic parameters – PwD and caregiver’s age, and cohabitation – and the remaining one a widely used measure in research and intervention with informal dementia caregivers – the caregiver’s perceived burden measured with the Zarit Burden Interview. Both previous empirical work (e.g., [19, 20, 22, 59]), and conceptual frameworks (e.g., [4]), on the factors associated with or predictors of the caregiver’s desire to institutionalize have been considering a plethora of variables pertaining to the caregivers, the PwD, and their context, often proposing complex models. This study departed from a comprehensive set of variables and managed to produce a simplified model. The simplicity of the derived rules makes it easy and promising to be applied to other datasets of informal dementia caregivers, which most certainly include the sociodemographic variables on this model, and often appraise burden with the same scale used in this research. Given that the desire to institutionalize is evidenced as the most important predictor of actual institutionalization [4, 24–27], this model may prove useful in predicting actual placement, which will be further investigated by these authors in the context of longitudinal research (see Limitations and future research).

### Limitations and future research

The findings of this study should be interpreted within the context of its limitations to provide a more comprehensive understanding of the results.

Regarding the recruitment method, participants in this study were not randomly selected; rather, they were enrolled after registering on the eHealth program iSupport-Portugal. iSupport-Portugal was promoted through various channels, including collaboration with community projects and services, patient associations, communication with health administrations and services, and engagement with practitioners in both private and public practice. Nevertheless, the recruitment method may indeed elevate the probability of volunteer bias. It is reasonable to anticipate that informal caregivers seeking training and support resources, such as iSupport, may actively strive to enhance their caregiving capabilities to continue providing care at home. As a result, registered users on iSupport-Portugal may be less inclined towards institutional placement of the person with dementia. On the other hand, participation in this research was exclusively online, enrolling digitally literate caregivers. Digital literacy is associated with younger age (with influential factors as e.g., digital nativity and ageism [73]), urban residency, and higher education [74], and has been highlighted as a “super social determinant of health” [75]. Indeed, there appears to be an overrepresentation of employed and highly educated caregivers in this sample, averaging 15 years of schooling. This level of education is higher than that described in previous national studies involving informal dementia caregivers [76]. However, clear, and comprehensive national statistics on the characteristics of caregivers are currently unavailable. Moreover, this study may have been able to recruit a subset of caregivers with specific characteristics related to education, employment status, and the relationship with the PwD (mostly children) that are not typically accessible through conventional community services. For example, employed caregivers may face accessibility issues, and such individuals are often underrepresented in studies using conventional recruitment methods [77]. Nonetheless, the study sample is diverse and, apart from the aforementioned variables, exhibits relatively typical sociodemographic characteristics (e.g., a high rate of female caregivers), caregiving context (e.g., a high rate of cohabitation), and caregivers’ psychological needs (e.g., a high perceived burden). As political investments in closing the digital divide begin to yield results and digital natives assume the role of caregivers, the diversity of iSupport-Portugal users may increase. This expansion could further enhance its utility as a tool for data collection on dementia care dyads.

Another limitation is that all data were collected remotely and relied on self-report from participants regarding the characteristics of care recipients. Consequently, a formal diagnosis of dementia for their care recipient was not confirmed in a clinical setting. This limitation reflects one of the trade-offs associated with collecting nationwide data in a cost-effective manner permitted by digital platforms, all of which aim to facilitate timely research.

Regarding data collection methods, while Module 0 on iSupport-Portugal (see Introduction) was deliberately designed to capture a comprehensive set of variables associated with caregiver and PwD outcomes, including institutional placement, it has resulted in a relatively time-consuming protocol. Roughly 40% (37.6%) of registered caregivers in the eHealth program who consented to participate in research have not completed the DIS and were therefore not included in this study. Another potentially contributing factor for dropout is the fact that the mobile version of iSupport-Portugal is still under improvement, which may impact the convenience and usability of self-completion measures. Dropouts in completing the measures, while not uncommon in internet research, could potentially be reduced by trimming Module 0 and enhancing usability, accessibility, and notification features of iSupport-Portugal.

These issues may have contributed to the not-so-large sample size in this study, despite previous research on the desire to institutionalize a PwD typically enrolling relatively modest sample sizes (e.g., [19, 23]). Informal caregivers of PwD often face numerous demands and are more likely to experience psychological distress, making it challenging to enrol and retain these participants in both interventions and research [77]. For the predictive model, the number of cases did not allow for splitting the dataset into testing and training sets for constructing the model and performing cross-validation. Therefore, confidence intervals for all metrics were calculated using bootstrapping to convey the uncertainty of the predictions. Overall, the confidence intervals for the model’s performance metrics suggest low uncertainty in the estimations (e.g., model accuracy of 0.83, with a 95% CI ranging from 0.75 to 0.89). It must be noted that iSupport-Portugal is a live platform that will continue to receive new data, possibly allowing for the validation of the derived model in future cases.

Upcoming research aims to use iSupport-Portugal to monitor a cohort of registered caregivers over a 9-month period, tracking the actual institutionalization of the PwD under their care. While caregivers’ desire to institutionalize is deemed the strongest predictor and a precursor of actual placement, longitudinal research is necessary to assess the predictive ability of the DIS regarding actual institutionalization, and whether predictors of institutionalization desire also forecast actual placement. Even though a cohort study by the authors is ongoing to this end, the extent of loss to follow-up and the rate of outcome occurrence remains to be determined.

Other forthcoming endeavors include the development of an add-on module to iSupport-Portugal aimed at addressing the topic of informal care discontinuation due to the institutionalization of the PwD. The need of accessing training and support was expressed by informal caregivers in previous national research [78]. The add-on module, co-created with end-users and based on previous literature, should be examined for its impact on modifiable factors linked to increased PwD institutionalization risk, such as caregiver burden. Subsequently, its influence on both the desire to institutionalize and actual institutionalization of PwD should be assessed.

## Conclusions

This paper innovatively incorporates a comprehensive array of sociodemographic, clinical, psychosocial, and contextual variables, gathered remotely via an eHealth platform, to construct a predictive model on informal dementia caregivers’ desire to institutionalize the PwD. The resulting analysis yielded a simplified, interpretable, and accurate model, primarily anchored in the caregiver’s perceived burden, and including caregiver and care recipient age, as well as cohabitation status as predictors.

The decision to place a relative with dementia in an institutional setting represents a critical life event, demanding significant behavioral, cognitive, and emotional adjustments from caregiving dyads. Effectively understanding and predicting this event, along with its antecedent - the desire to institutionalize - are crucial for intervening in modifiable factors, notably caregiver psychological distress, with the goal of delaying institutional placement. Furthermore, such insights are pivotal for enabling effective care planning and financing, while also mitigating the potential exacerbation or chronicity of stress experienced by informal caregivers as they transition away from their caregiving roles.

## Data Availability

Relevant raw data will be freely available to any researcher wishing to use them for non-commercial purposes, without breaching participant confidentiality, and upon request to the authors.
